# Professional learning using storytelling videos of childbirth experiences: A qualitative pilot study of student midwives' perceptions in Myanmar

**DOI:** 10.1111/jjns.12456

**Published:** 2021-09-28

**Authors:** Asako Noya, Michiko Oguro, Shigeko Horiuchi

**Affiliations:** ^1^ St. Luke's International Hospital Tokyo Japan; ^2^ Tokyo Healthcare University Chiba Japan; ^3^ St. Luke's International University Tokyo Japan

**Keywords:** birth experience, midwife, nursing education, patient voice, storytelling

## Abstract

**Aims:**

To explore and describe Myanmar student midwives' perceptions of professional learning using storytelling videos of women's childbirth experiences by conducting a qualitative study and to assess potential pedagogical uses of storytelling videos in midwifery education.

**Methods:**

This pilot study used a qualitative content analysis study design implemented as part of the Diversity and commonalities of birth from women's voice sharing project of the Toyota Foundation. Storytelling videos of the childbirth experiences of seven Asian women (three Myanmar, three Lao, and one Japanese women) were viewed by the students. Thereafter, a semistructured interview with focus group discussion was conducted to explore and assess the students' perceptions.

Setting: Kyaing Tong township, Shan State, Myanmar.

Participants: Five second‐year midwifery students from a single midwifery training school in Myanmar.

**Results:**

Data analysis of their perceptions yielded four major categories: (1) deep reverence for women/mothers;; (2) respectful attitude as a midwife to support mothers; (3) wish for a safe and secure birth environment; and (4) importance of learning from the mother's voice.

**Conclusions:**

The student midwives realized the importance of listening to the mothers' voices regardless of the nationalities or cultural background. The findings indicated that the storytelling videos broadened the students' perspectives of pregnancy and childbirth, and motivated them to provide better maternity care. Storytelling videos of childbirth experiences can be powerful educational materials for enhancing professional learning of student midwives.

## 1 INTRODUCTION

Childbirth is a physiological as well as a universal process among people across the world. The latest World Health Organization (WHO) guideline (2018) has identified “positive childbirth experience” as a significant endpoint for all women undergoing labor. The WHO has emphasized the importance of respectful maternity care, and the need for the attending staff and health services to create an enabling maternity care that encourages women's sense of control and involvement in decision‐making (Bohren et al., 2018; World Health Organization, 2018). Moreover, the WHO (2018) has indicated that respectful maternity care is an evidenced‐based critical component of providing safe and good‐quality care.

Disrespect and abuse (D&A), including abuse, neglect or disrespect during childbirth, is a violation of a woman's fundamental human rights (World Health Organization, 2014). The sad reality is that many women continue to experience D&A treatment during childbirth in health facilities. Thus, promoting respectful care has been increasingly recognized as a fundamental factor for improving the quality of maternity care. These aspects highlight the need and right of all women to have their voice heard during and following pregnancy and childbirth. As for D&A, there have been many reports from low‐ and middle‐income African and South Asian countries since 2015 (Bohren et al., 2015). However, reports from Southeast Asian countries remain limited.

Myanmar is one of the low‐ and middle‐income countries in Southeast Asia (The World Bank in Myanmar, 2020). Its maternity mortality rate is 282 deaths per 100,000 live births. This is the second highest rate among countries in the Association of Southeast Asian Nations (ASEAN), and is higher than the 140 deaths in Southeast Asian countries, 230 in developing countries, 216 in countries across the world, and 16 in developed countries (United Nations Economic Commission for Europe, 2017). The under‐five mortality rate of Myanmar is 50 deaths per 1,000 live births, and the neonatal mortality rate is 25 deaths per 1,000 live births (Taw, 2017). Despite the decline of these three indicators over the last decade, Myanmar has not yet fully met Millennium Development Goals four and five. Moreover, more than half of the overall births in Myanmar were delivered at home (63%) compared with births delivered in health facilities (37%) (Taw, 2017). Skilled providers including nurses, midwives, and doctors have provided assistance to 60% of the overall births. Traditional birth attendants have assisted in 29% of births, auxiliary midwives in 6%, and relatives or friends in 4% (Taw, 2017). To increase the quality of nursing and midwifery education programs, the Myanmar Nurse and Midwife Council (MNMC) published the *Standards and Criteria for Accreditation of Nursing and Midwifery Education Programs in Myanmar* in 2015. This document describes the professional expectations and criteria for measuring and evaluating professional performance (The Republic of the Union of Myanmar Nurse and Midwife Council, 2015). However, insights into how to improve the quality of respectful care by midwifes during their professional education remain unclear. Importantly, reports on the educational experiences of learning from women's voices or stories remain lacking.

Previous studies have indicated that listening to patients' stories can be a valuable strategy for promoting the learning process, and it may have a positive impact on delivering patient‐centered care (Christiansen, 2011; Gidman, 2013; Herxheimer et al., 2000; Lee & Lamp, 2005). Davidhizar and Lonser (2003) found that classroom storytelling was beneficial to “enhance self‐esteem”, “develop critical thinking”, “teach ethics”, “teach cultural sensitivity”, “provide role modeling”, and “teach communication”. Students felt that stories helped them retain information better (Garner, 2016).

Although most studies have focused on stories of patients who had illnesses or handicaps, some have indicated the outcomes from listening to childbirth stories. Lee and Lamp (2005) reported that birth storytelling enhanced students' appreciation of the personal meaning of pregnancy and childbirth. They also suggested that stories help students make progress in recognizing their role identity as nurses and highlighted the importance of considering the influence of healthcare providers on the life experiences of others (Lee & Lamp, 2005). Storytelling as a learning material in the classroom has taken various forms such as direct storytelling from patients, and using text words, journals, simple audios, audio with soundtracks, or videos (Christiansen, 2011; Gidman, 2013; Lee & Lamp, 2005; Waugh & Donaldson, 2016). Herxheimer et al. (2000) described that listening to real patient's stories is a valuable experience to help students understand the patients' perspectives.

In fact, there are several websites disseminating storytelling videos of personal experiences related to health and illness. These websites mainly provide patients' stories with videos, images, and music with a text format. These include the UK‐based Database of Individual Experiences (DIPEx) and the US‐based Patient Voices Program. Brown et al. (2008) concluded that a paradigm shift has been transforming nursing education from passive learning to a more active self‐directed, participative learning. The nursing faculty are shifting from a teacher‐dominated to a learner‐centered teaching approach (Brown et al., 2008).

Nevertheless, most previous studies were conducted in high‐income countries. Hence, additional studies on clarifying students' experiences of learning by listening to women's voices or stories from low‐ and middle‐income countries are needed. Moreover, given the scarcity of evidence, the current basis of education provided by educators to midwifery students mostly relies on their clinical experiences. Thus, the aim of this study was to explore and describe the perceptions of Myanmar student midwives of professional learning using storytelling videos of women's childbirth experiences, and to assess potential pedagogical uses of storytelling videos in midwifery education.

## 2 METHODS

### 2.1 Research design and setting

This pilot study used a qualitative content analysis study design implemented as part of the Diversity and commonalities of birth from women's voice sharing project of the Toyota Foundation (Arimori, 2016). One of the researchers (MO) has established a good relationship with local coordinators throughout the professional midwifery organization in Kyaing Tong township, Shan State, Myanmar. Thus, this area was selected for the study to ensure optimal data collection. Our particular local coordinator helped us to select the nursing school that was most eligible to our research objectives and readily accessible. Kyaing Tong is about 456 km away from Taunggyi, the capital city of Shan State, and lies in the valley between the high mountains of the Shan Plateau and the Mekong River. The city is inhabited by the Wa, Shan, Akha, Palaung, and Lahu ethnic tribes. The literacy rate in Shan State is 64.6%, which is lower than the Union literacy rate of 89.5%. The literacy rate of men (70.3%) is higher than that of women (59.4%) (Ministry of Immigration and Population with technical support from UNFPA, 2015).

### 2.2 Participants

The participants were second‐year midwifery students. They were chosen as they already have some clinical training experience compared with first‐year midwifery students. Specifically, the inclusion criteria were second‐year midwifery students who have experienced clinical training at a home or facility setting, have never administered childbirth, and speak the Myanmar language as the mother tongue. The exclusion criteria were second‐year midwifery students who have neither clinical training experience nor means of transport, and who are illiterate in the Myanmar language. Ethnicity was not a consideration and thus not included in the criteria.

Our local coordinator, who was originally from Myanmar, explained our research outline to the principal of the participating midwifery training school. Five eligible midwifery students agreed to participate. The researchers (AN, MO) explained the study details and interview process to the five students. They also obtained written informed consent for participation in the focus group discussion (FGD) and audio recording.

### 2.3 Data collection

The interviews consisted of semistructured questions with FDG. At the start of the discussion, an abridged version of storytelling videos (34 min) about the childbirth experiences of three Myanmar, three Lao, and one Japanese women were played. The original videos were made under the Diversity and commonalities of birth from women's voice sharing project (Arimori, 2016) and recorded in Japan, Myanmar, and Lao from December 2016 to March 2017. The women from these three countries were interviewed about their childbirth experiences from which storytelling videos were created. The present study used the abridged version of the videos from the three countries as women's voices and stories. The videos showed the women's responses to three questions. (1) Tell us about your pregnancy and childbirth. (2) If you have another opportunity to give birth, what is your ideal birth? (3) What is your definition of a “happy birth”?

After watching the videos, a 90‐min FGD was conducted by the researchers (AN, MO). The FGD questions were carefully developed and agreed upon by the research team according to the responses and questions in the videos, and considering existing evidence (Vaughn et al., 1996). The questions were as follows. (1) How did you feel after watching the videos? (2) Which scenes did you feel were closely related to you and why? (3) Which stories or statements impressed you the most and why? (4) Did the videos change your perspectives or thoughts about childbirth? (5) Do you have any questions or are there any unclear parts about the video contents? (6) How did you feel about women's hopes for birth? (7) Do you think is it easy to give birth? (8) With whom do you want to share these videos and why? All the responses were faithfully translated into Japanese. During the FGD, the researchers (AN, MO) took field notes and audio recordings, and then transcribed the contents verbatim for analysis. The FGD was conducted with the support of an interpreter who was fluent in Myanmar, Shan, and Japanese. Data were obtained on August 29, 2018.

### 2.4 Data analysis

Data were analyzed inductively following the five‐stage process of Vaughn et al. (1996) as follows. The first stage involved creating the verbatim data from the recording and reading the entire description to get a sense of the participant's thoughts. The second stage consisted of identifying the meaning units. The third stage involved categorizing the central theme within each meaning unit. The fourth stage included identifying the meaning units in terms of the phenomenon of interest, that is, the impacts of storytelling. The fifth and final stage involved synthesizing the transformed meaning units into general statements of the subject's experience. The researchers maintained scientific rigor in this study by engaging in continuous discussion among themselves throughout the whole research‐analysis process.

After completing the FGD, the researchers listened to the audio recordings and created a verbatim transcript. The transcript was read in its entirety to gain a good sense of the students' thoughts. Meaning units were identified and the central theme within each meaning unit was subsequently divided into subcategories which were similarly categorized to create a larger abstraction of categories (Vaughn et al., 1996). The researchers‐academic supervisors (MO, SH), who have extensive experience in qualitative and multicultural studies, checked all the data and maintained the scientific rigor of this study by continuous consultation and discussion throughout the research‐analysis process. As for qualitative validation, we used member‐checking, triangulation of the data from transcripts and observational notes and compared these with disconfirming evidence (Creswell & Clark, 2011).

### 2.5 Ethics statement

Ethical approval was obtained from the University of Tokyo Healthcare Research Ethics Committee (KYO30‐17B). Before the FGD, verbal and written consents for data reporting were obtained from the midwifery students who met the inclusion criteria and agreed to participate. They were also informed of their right to confidentiality and to withdraw from the study at any point in time without penalty. The consent form was written in Myanmar and explained by our local coordinator.

## 3 RESULTS

### 3.1 Participants' background

Five second‐year undergraduate midwifery students were recruited from a midwifery training school in Myanmar, which has a total of 60 students. Their ages were between 20 and 23 years (mean 21, *SD* 1.22). All of them had not yet experienced administering childbirth. Their ethnicities were Akha (n = 2), Burmese (n = 1), Lahu (n = 1), and Shan (n = 1). These ethnic minority students were not chosen intentionally. Those who agreed and participated just happened to be ethnic minority students. During their home or clinic setting practicum, each student experienced providing support to one to three childbirths. The characteristics of each participant are shown in Table 1. In Myanmar, midwives are regarded as general health workers. Therefore, they are the main service providers for maternal and reproductive health at the community level. All of them plan to work at rural health centers after graduation.

**TABLE 1:** Characteristics of participants

	Ethnicity, religion	Age (years)	Practicum experience	Grade in midwifery training school
A	Burmese, Buddhist	20	Two home births	Second year
B	Akha, Christian	21	Two clinic births	Second year
C	Akha, Christian	20	Three clinic births	Second year
D	Lahu, −	23	Three clinic births	Second year
E	Shan, Buddhist	21	One home birth	Second year

Data analysis yielded four major perception categories: (1) deep reverence for women/mothers; (2) respectful attitude as a midwife to support mothers; (3) wish for a safe and secure birth environment; and (4) importance of learning from the mother's voice.

### 3.2 Thematic categories and subcategories from students' FGD

#### 3.2.1 Deep reverence for women/mothers

This category encompasses various profound feelings for women who have the possibility to be a mother (Table 2). It is divided into three subcategories: (a) impressed by the mother's love; (b) marveled by the diversity of the mother's perspectives and childbirth experience; and (c) honored and proud to be a woman.

**TABLE 2:** Thematic categories and subcategories from students' focus group discussions

Categories	Subcategories
(1) Deep reverence for women/mothers	(a) Impressed by the mother's love (b) Marveled by the diversity of the mother's perspectives and childbirth experience (c) Honored and proud to be a woman
(2) Respectful attitude as a midwife to support mothers	(a) Provide considerate care for mothers to know more about their feelings (b) Reconsider the stance as a midwife to support mother's decision between home birth and facility birth
(3) Wish for a safe and secure birth environment	(a) Strong recommendation for facility birth together with medical professionals (b) Realization of the necessity to create a safe and supportive environment for all women
(4) Importance of learning from the mother's voice	(a) Clarification of a desire for future learning (b) Recognition of women's real voices as being unforgettable

##### Impressed by the mother's love

By listening to the women's stories, the students were deeply moved by the mother's love. They felt the mother's love when the mothers overcame the difficult labor pain and could even think about their next childbirth. The students were amazed at how the mothers were able to share what makes them happy about their birth experience.

*“I felt (that) these seven mothers were all filled with great love*.*”*

*“I respect the mothers because even though childbirth was tough*, *they could enjoy their experience*. *They could also think about how they want to give birth and what makes them happy for their own childbirth*.*”*

##### Marveled by the diversity of the mother's perspectives and childbirth experience

With the wide views and various childbirth stories of the women, the students conveyed their admiration and curiosity of how the women drew their valuable thoughts.

*“I was very impressed by one of the women in the video because she attended prenatal school and prepared for a long time for her birth*. *Besides*, *she got rid of her concern about birth by listening to other women's voice*.*”*

*“I wonder who taught the ethnic minority women the idea that giving birth by themselves is a natural way*. *The more people watch*, *the longer time it takes*. *Babies are too shy to come out if other people watch them during birth*. *I was also surprised and impressed that she told us she was not afraid of labor pain at all*, *because she knew it was a natural process and the baby could not be born without it*.*”*

##### Honored and proud to be a woman

Watching and listening to the women's stories changed the students' perceptions of being a woman.

*“I'd been thinking that being a woman was too much work for me*. *But after hearing (one of the women in the video say) ‘only women can experience childbirth’*, *I again realized that I appreciate being a woman*.*”*

#### 3.2.2 Respectful attitude as a midwife to support mothers

This category consists of two subcategories about midwifery support for mothers, namely: (a) provide considerate care for mothers to know more about their feelings; and (b) reconsider the stance as a midwife to support mother's decision between home birth and facility birth.

##### Provide considerate care for mothers to know more about their feelings

By making self‐realizations based on the women's perspectives, the students reconsidered their concept of midwifery care and became more motivated to provide intimate and compassionate care for mothers. They were inspired to take another look at the hope and satisfaction of the women from giving birth.

*“I want to provide mothers not only knowledge of childbirth*, *but also compassionate care*, *which is enough to make them want to visit the hospital*.*”*

*“I want to consider how I can support mothers to make their childbirth happy and satisfying besides ensuring safety of their lives*.*”*

##### Reconsider the stance as a midwife to support mother's decision between home birth and facility birth

This subcategory explains the importance of continuous support by medical professionals throughout the whole term of pregnancy and childbirth, a specific support plan for each woman's birthplace of choice, and midwives' own preparations.

*“At first*, *I will confirm the mother's choice for the place of birth when I hear the news of pregnancy for the first time*. *If some mothers choose home birth*, *I want to explain both disadvantages and advantages with their choice and tell them to attend the prenatal check on a regular basis*. *Finally*, *I want them to understand that they can choose home birth only when there are no abnormalities in the mother and baby*.*”*

*“I will keep in mind to make all the preparations for supporting home birth*.*”*

#### 3.2.3 Wish for a safe and secure birth environment

This category was structured into two subcategories: (a) strong recommendation for facility birth together with medical professionals; and (b) realization of the necessity to create a safe and supportive environment for all women.

##### Strong recommendation for facility birth together with medical professionals

Despite efforts to promote institutional delivery, home delivery is still very common in Myanmar. In fact, about half of all live births are delivered at home. Moreover, two‐fifths of all births are not assisted by skilled providers such as nurses, midwives, and doctors.

*“I really want mothers to give birth in a hospital for their safety*. *Because home births might be unclean or may lack resources*. *In addition*, *owing to the bad condition of the roads or the long distance to travel*, *it can be too late if they start to transfer to a hospital after something already happened to the mother or baby*.*”*

##### Realization of the necessity to create a safe and supportive environment for all women

From the women's stories, the students realized the situations that acted as barriers and prevented women from having a safe childbirth environment. These included inconsiderate care at hospitals, inaccessibility of health facilities, and restrictions from cultural backgrounds. These situations made the students realize the necessity to create a safe and supportive environment for all women.

*“I think the inconsiderate care of medical professionals was one of the reasons why everyone did not want to give birth in a hospital*.*”*

*“I strongly hope Myanmar will become one of the countries where most childbirths occur at a hospital or a clinic where women can receive continuous care*.*”*

#### 3.2.4 Importance of learning from the mother's voice

This category consisted of two subcategories: (a) clarification of a desire for future learning; and (b) recognition of women's real voices as being unforgettable.

##### Clarification of a desire for future learning

The students were interested in knowing the situations of pregnancy and childbirth in other countries. They were motivated to know more about the need for physical and mental support by women during childbirth. They realized that they have not been taught about providing emotional support during labor.

*“I want to know more details about prenatal training* [education] *programs and how the medical staff manage these programs in other countries*.*”*

*“In order to work in the actual medical field*, *I want to learn more about what kinds of support do mothers want us* [medical professionals] *to do for them*.*”*

##### Recognition of women's real voices as being unforgettable

The students wanted to share the videos with others because they recognized women's real voices as being unforgettable for clinical midwives, student midwives, and other pregnant women. The students felt that the women's voices helped them to better understand the feelings of women undergoing labor. The women's voices gave them a chance to reconsider their concept of midwifery care. The students also thought that the women's stories would help other women to make decisions about pregnancy and childbirth.

*“I eagerly want to share these videos with clinical midwives and student midwives*. *The reasons are that these videos made us realize women's perspectives during labor even if we have never experienced administering childbirth ourselves*. *Also*, *the videos gave us the chance to reflect on our concept of midwifery care*, *and they motivated us to dispel women's fear and ease their loneliness during childbirth*.*”*

*“I think watching these videos will help pregnant women to know other women's choices and experiences*. *And the videos will encourage them to reconsider their own choices*.*”*

## 4 DISCUSSION

### 4.1 Women's childbirth stories evoke respect for women and mothers

The students' profound impression and high respect for women were inspired by the insights they gained from the women's feelings during the storytelling. Respecting women is essential particularly during the pregnancy and childbirth periods in accordance with the WHO statement which states that “every woman has the right to the highest attainable standard of health, which includes the right to dignified, respectful healthcare throughout pregnancy and childbirth, as well as the right to be free from violence and discrimination” (World Health Organization, 2014). Disrespectful and abusive treatment of women during childbirth in facilities is a public health problem that violates the fundamental rights of women and the unborn child. This remains a burning issue across the world. The WHO advocates respectful maternity care in accordance with a human rights‐based approach to reducing maternal morbidity and mortality. This could improve women's experience of labor and childbirth and address health inequalities (World Health Organization, 2018). Although the situation of D&A remains unclear in Southeast Asia, Maung et al. (2020) described that differences in access to resources (e.g., financial resources), information about pregnancy and childbirth, and support from family members during labor might impact how women are treated. Furthermore, social norms around pregnancy and childbirth, and relationships between healthcare providers and women shape women's experiences. Our findings in Myanmar are in agreement with those of Maung et al. (2020), and also emphasize them.

Having respect for women is fundamental to healthcare professional students. The present findings indicate that the storytelling videos of the women about their childbirth experiences touched the hearts of the students and inspired them to have a deep reverence for mothers/women. We consider that the narrative stories of the individual mothers can serve as essential educational materials.

Several studies are in agreement with our findings. Christiansen (2011) noted that patients' stories could attract students' attention, stimulate their interest, and draw them into another world. Gidman (2013) reported that listening to patients' stories during practice placements provided a valuable alternative source of knowledge for students, particularly with respect to understanding patients' perspectives. Lee and Lamp (2005) found that women's stories could help students more fully appreciate pregnancy and childbirth. In turn, this appreciation can serve as a foundation for building their professional commitment to holistic and client‐centered care. Hence, listening to mothers' and women's stories is considered as key factors in putting respectful maternity care into practice and in building a professional commitment to women‐centered care.

### 4.2 Midwives' attitudes and support for decision‐making regarding the place of birth are vital to ensuring women‐centered care

The students' increased knowledge of the importance of midwives' attitudes and care from the storytelling videos was reflected in their realization of the need to have a more respectful attitude as a midwife to support mothers (category 2) and in their wish for a safe and secure birth environment (category 3). Myanmar has a maternity mortality rate of 282 deaths per 100,000 live births, which is the second highest among ASEAN countries (Department of Population, Ministry of Labor, Immigration and Population, 2016). Home birth remains popular, especially in rural areas and among women with less than a secondary education level (Taw, 2017). The Women's Organization Network showed that both cost and distance prevent many women from seeking the healthcare that they desperately need (Women's Organization Network [WON], 2016). Most of the students suggested facility birth because of its safety. Understanding the diversity of women's thoughts and experiences, specifically about the decision of the place of birth, made the students realize the necessity to create a safe and supportive environment for all women (category 3b). Nevertheless, the students indicated that they will support the mothers' choice of birthplace whether it be the facility or home. They also expressed their commitment to provide continuous support and a safe environment for all women.

### 4.3 The chance to learn about providing emotional support is not available

By understanding the mother's fear, loneliness, and dissatisfaction about hospital care from the storytelling videos, the students felt that it is important to provide considerate care for mothers to know more about their feelings (category 2a). The students thought that one of the reasons why the women did not want to give birth in a hospital was because of the inconsiderate care of medical professionals. Watson et al. (2020) reported that a woman's expectations or apprehensions of birth can be damagingly impacted by negative healthcare provider's interactions which can leave women feeling dismissed and disregarded. They concluded that a lack of education and support limited informed decision‐making, resulting in feelings of losing control and powerlessness which contribute to women's trauma. The International Confederation of Midwives advocates the following aspects of care: “care during labor and birth; assess woman's physical and behavioral responses to labor; provide information, support, and encouragement to women; and support persons throughout labor and birth” (International Confederation of Midwives, 2019). Thus, the storytelling videos made the students realize the need of mothers for emotional care during the pregnancy and childbirth periods.

### 4.4 Storytelling videos of childbirth experience is a powerful learning tool

The storytelling videos made the students realize the importance of learning from the mother's voice (category 4). After understanding the diversity of childbirth experiences, the students were interested in learning more about the situations in other countries. The students suggested that the storytelling videos might be helpful for peer women in terms of knowing the choices and experiences of other women and then making or reconsidering their own choices. Thus, the storytelling videos gave the students new perspectives and motivated them to learn more from the mothers' voices. Roberts et al. (2021) also concluded in their study that media clips could be used to open up classroom discussions, provide some access to uncommon issues that they may not see in practice during their training, and support visual learners. Additionally, as one of the examples of using digital resources as a learning tool, O'Connor et al. (2020) provided the synthesized evidence of podcasting in nursing and midwifery education. They identified various podcasting interventions and their applications to nursing and midwifery training which may benefit both educators and students.

The UK‐based DIPEx showed that the qualitative analysis of patients' perspectives of illness could illuminate numerous important issues. It is therefore an innovative resource for medical education, with some contents unlikely to arise in a clinical encounter (Herxheimer et al., 2000). Christiansen (2011) described that patient digital stories are powerful learning tools that offer students an opportunity to transcend their own personal frame of reference and engage with the reality of others. Through a process of defining the meaning, emotional engagement, and reflection, students can generate new insights that can potentially transform their developing sense of professional identity (Christiansen, 2011).

Taken together, Gidman (2013) so correctly captures the essence of patient stories when he stated that “educators need to recognize and value learning from patient stories, and it requires a patient and student‐centered approach to learning and an understanding of storytelling as a learning and teaching strategy” (p. 196). Our findings are consistent with Gidman's statement, and indicate that stories of real‐life experiences of patients, mothers, or women are effective tools for cultivating learners.

Overall, the present findings added the following valuable insights to existing knowledge, emphasizing the importance of this study: listening to stories of mothers and women places respectful maternity care into practice and builds a professional commitment to women‐centered care; comprehending the diverse thoughts and experiences of women (e.g., place of birth decision) motivates the creation of a safe and supportive environment for women; watching storytelling videos leads to a realization of the need for emotional care for mothers during pregnancy and childbirth; and hearing stories of real‐life experiences serves as an effective tool for cultivating learners.

### 4.5 Research limitations and future perspectives

In this pilot study, the sample size of the participating students was small, and the students were from specific ethnic groups selected from second‐year students in one midwifery training school. Therefore, the school's culture and curriculum may have similarly influenced their opinions or views. As the students were from different ethnic groups within Myanmar, some may have withheld their opinions on a certain issue owing to their ethnic background. Although our FGD included only five participants, the obtained results provided rich and well‐saturated data. This indicated that the number of participants was sufficient to meet the aims of this study. Moreover, the findings of this study may be generalized to the student midwives as per participants of this study.

In future studies, we will aim to incorporate other materials on narrative childbirth stories such as those found in DIPEx and other resources from other countries.

## 5 CONCLUSIONS

This pilot study assessed professional learning using storytelling videos of women's childbirth experiences by conducting a qualitative study of the perceptions of student midwives in Myanmar. Four major perception categories were identified: (1) deep reverence for women/mothers; (2) respectful attitude as a midwife to support mothers; (3) wish for a safe and secure birth environment; and (4) importance of learning from the mother's voice. The storytelling videos enhanced the students' professional learning as follows. First, they made the students realize the importance of having respect and a positive impression of women and mothers. Second, they clarified the correct attitudes of midwives, particularly in terms of providing support in decision‐making regarding the place of birth between facility and home. Third, they helped the students recognize the need of mothers for emotional care during the pregnancy and childbirth periods. Fourth, they provided the students with new perspectives and motivated them to learn more about pregnancy and childbirth. Taken together, storytelling videos of childbirth experiences can be powerful education materials for enhancing the professional learning processes of student midwives.

## CONFLICT OF INTEREST

The authors declare they have no conflicts of interest associated with this study.

## AUTHOR CONTRIBUTIONS

This study was conducted as part of a research project of Professor Michiko Oguro under the Toyota Foundation 2016 International Grant Program (PI: Tokyo Healthcare University). Asako Noya (St. Luke's International Hospital) and Michiko Oguro conceptualized and designed the study, collected data, and drafted the manuscript. Asako Noya, Michiko Oguro, and Shigeko Horiuchi (St. Luke's International University) performed the data analysis. Shigeko Horiuchi made important content revisions and refined the manuscript content. All authors read and approved the final manuscript.

## References

[jjns12456-bib-0001] Arimori, N. (2016). Diversity and commonalities of birth from women's voice sharing. Retrieved from The Toyota Foundation International Grant Program. https://toyotafound.secure.force.com/psearch/JoseiDetail?name=D16-N-0128

[jjns12456-bib-0002] Bohren, M. A. , Vogel, J. P. , Fawole, B. , Maya, E. T. , Maung, T. M. , Baldé, M. D. , Oyeniran, A. A. , Ogunlade, M. , Adu‐Bonsaffoh, K. , Mon, N. O. , Diallo, B. A. , Bangoura, A. , Adanu, R. , Landoulsi, S. , Gülmezoglu, A. M. , & Tunçalp, Ö. (2018). Methodological development of tools to measure how women are treated during facility‐based childbirth in four countries: Labor observation and community survey. BMC Medical Research Methodology, 18(1), 132. 10.1186/s12874-018-0603-x 30442102 PMC6238369

[jjns12456-bib-0003] Bohren, M. A. , Vogel, J. P. , Hunter, E. C. , Lutsiv, O. , Makh, S. K. , Souza, J. P. , Aguiar, C. , Saraiva Coneglian, F. , Diniz, A. L. , Tunçalp, Ö. , Javadi, D. , Oladapo, O. T. , Khosla, R. , Hindin, M. J. , & Gülmezoglu, A. M. (2015). The mistreatment of women during childbirth in health facilities globally: A mixed‐methods systematic review. PLoS Med, 12(6), e1001847; discussion e1001847. 26126110 10.1371/journal.pmed.1001847 PMC4488322

[jjns12456-bib-0004] Brown, T. S. , Kirkpatrick, K. M. , Mangum, D. , & Avery, J. (2008). A review of narrative pedagogy strategies to transform traditional nursing education. Educational Innovations, 47(6), 283–286. 10.3928/01484834-20080601-01 18557318

[jjns12456-bib-0005] Christiansen, A. (2011). Storytelling and professional learning: A phenomenographic study of students' experience of patient digital stories in nurse education. Nurse Education Today, 31(3), 289–293. 21074909 10.1016/j.nedt.2010.10.006

[jjns12456-bib-0006] Creswell, J. W. , & Clark, V. L. P. (2011). Designing and conducting mixed methods research (2nd ed.). Sage Publication Inc.

[jjns12456-bib-0007] Davidhizar, R. , & Lonser, G. (2003). Storytelling as a teaching technique. Nurse Educator, 28(5), 217–221. 14506353 10.1097/00006223-200309000-00008

[jjns12456-bib-0008] Department of Population, Ministry of Labour, Immigration and Population . (2016). The 2014 Myanmar Population and Housing Census Thematic Report on Maternal Mortality (Census Report Volume 4‐B). https://myanmar.unfpa.org/sites/default/files/pub-pdf/4B_Mortality.pdf

[jjns12456-bib-0009] Garner, S. L. (2016). Patient storytelling in the classroom: A memorable way to teach spiritual care. Journal for Christian Nursing, 33(1), 30–34. 26817368

[jjns12456-bib-0010] Gidman, J. (2013). Listening to stories: Valuing knowledge from patient experience. Nurse Education in Practice, 13(3), 192–196. 23084838 10.1016/j.nepr.2012.09.006

[jjns12456-bib-0012] Herxheimer, A. , McPherson, A. , Miller, R. , Shepperd, S. , Yaphe, J. , & Ziebland, S. (2000). Database of patients' experiences (DIPEx): A multi‐media approach to sharing experiences and information. Lancet, 355(9214), 1540–1543. 10801187 10.1016/S0140-6736(00)02174-7

[jjns12456-bib-0013] International Confederation of Midwives . (2019). Essential competencies for midwifery practice 2019 UPDATE. https://internationalmidwives.org/our‐work/policy‐and‐practice/essential‐competencies‐for‐midwifery‐practice.html

[jjns12456-bib-0015] Lee, J. C. , & Lamp, K. J. (2005). The birth story interview: Enhancing student appreciation of the personal meaning of pregnancy and birth. Nurse Educator, 30(4), 155–158. 16030451 10.1097/00006223-200507000-00007

[jjns12456-bib-0016] Maung, T. M. , Show, K. L. , Mon, N. O. , Tuncalp, O. , Aye, N. S. , Soe, Y. Y. , & Bohren, M. A. (2020). A qualitative study on acceptability of the mistreatment of women during childbirth in Myanmar. Reproductive Health, 17, 56. 32312305 10.1186/s12978-020-0907-2 PMC7171855

[jjns12456-bib-0017] Ministry of Immigration and Population with technical support from UNFPA . (2015). 2014 Myanmar Population and Housing Census: A Changing Population: Shan State Figures at a Glance May 2015. https://myanmar.unfpa.org/sites/default/files/pub-pdf/3M_Shan_Figures_ENG.pdf

[jjns12456-bib-0020] O'Connor, S. , Daly, C. S. , MacArthur, J. , Borglin, G. , & Booth, R. G. (2020). Podcasting in nursing and midwifery education: An integrative review. Nurse Education in Practice, 47, 102827. 10.1016/j.nepr.2020.102827 32763834 PMC7336128

[jjns12456-bib-0022] Roberts, J. , Bennett, B. , Slack, H. , Borrelli, S. , Spiby, H. , Walker, L. , & Jomeen, J. (2021). Midwifery students' views and experiences of birth on mainstream factual television. Midwifery, 92, 102859. 33129184 10.1016/j.midw.2020.102859

[jjns12456-bib-0024] Taw, N. P. , (2017). Public health statistics (2014‐2016), Ministry of Health and sports, Department of Public Health. https://mohs.gov.mm/Main/content/publication/public‐health‐statistics‐report‐2014‐2016

[jjns12456-bib-0025] The Republic of the Union of Myanmar Nurse and Midwife Council . (2015). Guideline on “Standards and Criteria for Accreditation of Nursing and Midwifery Education Programs in Myanmar”. https://vdocuments.site/the‐republic‐of‐the‐union‐of‐myanmar‐myanmar‐nurse‐and‐the‐republic‐of‐the‐union.html

[jjns12456-bib-0026] The World Bank in Myanmar . (2020). https://www.worldbank.org/en/country/myanmar/overview

[jjns12456-bib-0027] United Nations Economic Commission for Europe . (2017). Meeting the Sustainable Development Goals in Myanmar using data collected in the 2014 Census. Geneva, Switzerland: United Nations Economic Commission for Europe. https://unece.org/fileadmin/DAM/stats/documents/ece/ces/ge.41/2017/Meeting-Geneva-Oct/WP26_ENG.pdf

[jjns12456-bib-0028] Vaughn, S. , Schumm, S. J. , & Singub, J. M. (1996). Focus group interviews in education and psychology. SAGE Publications, Inc.

[jjns12456-bib-0029] Watson, K. , White, C. , Hall, H. , & Hewitt, A. (2020). Women's experiences of birth trauma: A scoping review. Women and Birth, 34, 417–424. 33020046 10.1016/j.wombi.2020.09.016

[jjns12456-bib-0030] Waugh, A. , & Donaldson, J. (2016). Students' perceptions of digital narratives of compassionate care. Nurse Education in Practice, 17, 22–29. 27038084 10.1016/j.nepr.2016.01.008

[jjns12456-bib-0031] World Health Organization . (2014). The prevention and elimination of disrespect and abuse during facility‐based childbirth: WHO statement. https://apps.who.int/iris/handle/10665/134588

[jjns12456-bib-0032] World Health Organization , (2018). WHO recommendations on antenatal care for a positive pregnancy experience: Summary. Geneva, Switzerland. https://www.who.int/publications/i/item/9789241549912 28079998

[jjns12456-bib-0033] Women's Organization Network (WON) , (2016). The Voices of Myanmar Women, Research Report in support of CEDAW Alternative Report. https://www.ecoi.net/en/file/local/1352074/1930_1484747696_int-cedaw-ngo-mmr-24237-e.pdf

